# A short review on emotion processing: a lateralized network of neuronal networks

**DOI:** 10.1007/s00429-021-02331-7

**Published:** 2021-07-03

**Authors:** Nicola Palomero-Gallagher, Katrin Amunts

**Affiliations:** 1 Institute of Neuroscience and Medicine (INM-1), Research Centre Jülich, 52425 Jülich, Germany; 2 C. & O. Vogt Institute for Brain Research, Heinrich-Heine-University, 40225 Düsseldorf, Germany; 3 Department of Psychiatry, Psychotherapy, and Psychosomatics, Medical Faculty, RWTH, Aachen, Germany

**Keywords:** Emotion, Brain networks, Limbic system, Asymmetry, Cingulate

## Abstract

Emotions are valenced mental responses and associated physiological reactions that occur spontaneously and automatically in response to internal or external stimuli, and can influence our behavior, and can themselves be modulated to a certain degree voluntarily or by external stimuli. They are subserved by large-scale integrated neuronal networks with epicenters in the amygdala and the hippocampus, and which overlap in the anterior cingulate cortex. Although emotion processing is accepted as being lateralized, the specific role of each hemisphere remains an issue of controversy, and two major hypotheses have been proposed. In the right-hemispheric dominance hypothesis, all emotions are thought to be processed in the right hemisphere, independent of their valence or of the emotional feeling being processed. In the valence lateralization hypothesis, the left is thought to be dominant for the processing of positively valenced stimuli, or of stimuli inducing approach behaviors, whereas negatively valenced stimuli, or stimuli inducing withdrawal behaviors, would be processed in the right hemisphere. More recent research points at the existence of multiple interrelated networks, each associated with the processing of a specific component of emotion generation, i.e., its generation, perception, and regulation. It has thus been proposed to move from hypotheses supporting an overall hemispheric specialization for emotion processing toward dynamic models incorporating multiple interrelated networks which do not necessarily share the same lateralization patterns.

## Introduction

The brain is characterized by a conspicuous structural asymmetry which is accompanied by a functional lateralization, i.e., the hemispheres are differentially specialized for certain tasks, and to some extent can function independently of each other within the scope of these tasks. Although the focus of the present mini-review is on the human brain, it must be noted that lateralization of brain function in the emotional domain is not restricted to humans, but constitutes a widespread phenomenon found throughout the animal kingdom (Güntürkün et al. 2020; Rogers and Vallortigara 2015; Vallortigara and Rogers 2020). Chimpanzees and several species of Old World monkeys display a right hemisphere dominance for both the perception and expression of emotions (Lindell 2013; Zhao et al. 2020). Giant pandas exhibit a leftwards lateralization in the processing of positive but not of negative stimuli (Liu et al. 2021). In dogs, positively and negatively valenced stimuli are associated with a higher amplitude of tail-wagging movements to the right and to the left side, respectively (Siniscalchi et al. 2013). Emotional reactions of bottlenose dolphins are stronger when negative stimuli are presented on their right than on their left side (Charles et al. 2021), and cuttlefish have a right eye preference for brightness matching associated with their camouflage abilities (Schnell et al. 2018). Finally, whereas honeybees show a bias to turn toward the scent of isoamyl acetate, an alarm pheromone, when it is presented on the right, and turn away from the source of this scent when presented on the left, they did not display an asymmetry in turning response to the odor of flowers on which they had been feeding prior to testing (Rogers and Vallortigara 2019).

The most obvious structural lateralization in the human brain, the protrusions of the left occipital and the right frontal pole beyond their contralateral counterparts, is so prominent, that it is associated with so-called petalia-impressions on the inner surface of the skull (Hadziselimovic and Cus 1966a; Hadziselimovic and Ruzdic 1966b). Reports on functional lateralization date back as early as 1861, when Broca associated a lesion in the third convolution of the left frontal lobe with the patient’s sudden loss of the ability to speak (Broca 1861a; b). Besides the richly documented asymmetry associated with speech production and language comprehension (Friederici and Gierhan 2013), lateralization has also been described for functions as varied motor control (Amunts et al. 1997; Sainburg et al. 2016; Sokolowska, 2021), visuospatial skills (Ciricugno et al. 2021; Vogel et al. 2003), and emotion processing (Demaree et al. 2005; Packheiser et al. 2021), to name only a few of many studies.

Structural asymmetry has been found in several regions that are involved in emotion processing, including the cingulate cortex, and related to behavior, psychopathology and illness, e.g., schizophrenia (Fujiwara et al. 2007). Another study suggested that differences in asymmetry of the anterior cingulate region may correspond with behavioral style, i.e., disposition to fear and anticipatory worry (Pujol et al. 2002), and lateralization has been discussed for emotion processing (Demaree et al. 2005).

Emotions are valenced mental responses to internal or external stimuli, which trigger visceromotor reflexes and modulate perception and cognition as well as physiological arousal, i.e., they induce feelings (Cabanac 2002; Ocklenburg and Güntürkün 2018). Thus, emotions enable adaptive behavior in response to specific events. Whereas Murphy and Zajonc (1993) postulated the existence of only two classes of emotion (i.e., positive and negative), Russell and Barrett (1999), Russell (2003), identified four “core affect” categories resulting from the blend of hedonic and arousal values (i.e., pleasure/good, displeasure/bad, activated/energized and sleepy/enervated), and Ekman (1992), Ekman et al. (1969) defined six discrete emotional states (i.e., basic emotions) based on the distinct facial expressions with which they are associated (i.e., anger, fear, sadness, enjoyment (or happiness), disgust and surprise), and each one of these basic emotions is associated with a specific neural network (Fusar-Poli et al. 2009b).

## Brain regions subserving emotion

Emotion processing involves the *coordinated* activation of *multiple* large-scale neuronal networks encompassing both cortical and subcortical brain regions to enable identification of the emotional significance of stimuli as well as the induction and modulation of affective states and emotional behaviors.

Building on early models in which the limbic system was though to constitute the anatomic basis of emotions (MacLean 1970; Papez 1937; Yakovlev 1948, 1968), our understanding of the brain regions involved in the processing of this higher cognitive function has matured to the present concept of integrated pathways of distributed neural networks which are connected by the limbic system (Arciniegas 2013; Catani et al. 2013; Lindquist et al. 2012; Mesulam 2000; Pessoa 2018; Rolls 2015). Mesulam (2000) proposed that the amygdala and hippocampus are interconnected epicenters of two large-scale integrated pathways which are differentially involved in the various components of emotion and overlap in the anterior cingulate cortex.

### The amygdala-centered network

The amygdala-centered network constitutes the neurobiological substrate for the integration of sensory input and emotional arousal to decode the significance of the stimulus for the organism, and includes the amygdala, areas of the olfactory, orbitofrontal, insular, anterior and midcingulate cortex as well as the ventral striatopallidum (Amaral et al. 1992; Catani et al. 2013; Geschwind 1965; Mesulam 2000).

The role of the amygdala is to assess a sensory stimulus on the basis of its intrinsic hedonic properties and possible association with other previously acquired primary reinforcers, as well as based on the organism’s current motivational state to determine its valence and modulate its neural impact on the organism to induce an adequate emotional state (Mesulam 2000). Fear is probably the emotion category most often associated with the amygdala, and was first described in the seminal work by LeDoux (1994). Although the amygdala appears to play a more extensive role in negatively valenced emotions, it is also significantly involved in the processing of positively valenced stimuli (Cunningham and Kirkland 2014; Hamann et al. 2002; Wang et al. 2017), and depressed patients show higher amygdala responses to negative stimuli and lower amygdala responses to positive stimuli than do healthy controls (Groenewold et al. 2013). A meta-analytic functional connectivity-based parcellation of the amygdala revealed three clusters comparable in shape and relative position with the cytoarchitectonically identified laterobasal, centromedial, and superficial nuclei groups (Amunts et al. 2005; Bzdok et al. 2013). Functional profiling of the three clusters showed the “laterobasal cluster” to be associated with coordinating high-level sensory input, the “centromedial cluster” to mediate attentional, vegetative, and motor responses, and the “superficial cluster” to be involved in the processing of olfactory stimuli (Bzdok et al. 2013).

The amygdala is able to integrate and process multimodal information, a primordial requisite for the modulation of higher cognitive functions such as emotional behavior, and this integrative function is subserved by its connectivity with numerous cortical and subcortical structures belonging to multiple functional systems. Furthermore, individual neurons in the amygdala not only respond to all types of unimodal sensory or viscerosensory stimuli, but also to multimodal sensory stimuli, to reward or punishment-related reinforcers, and to stimuli with a cognitive significance (Yilmazer-Hanke 2012). The primate amygdala is connected with primary/higher order unimodal areas belonging to all sensory systems, with multimodal areas of the orbitofrontal, anterior cingulate, insular, and temporal cortex, including the hippocampal complex (Aggleton et al. 1980, 2015; Aggleton and Saunders 2000; Amaral 1986; Amaral et al. 1992; Carmichael and Price 1995; Freese and Amaral 2005; Price 2003; Young et al. 1994). It is also connected with numerous subcortical structures, including the basal forebrain, thalamus, hypothalamus, periaqueductal central gray, and the peripeduncular nucleus (Aggleton et al. 1980; Price 2003). These connections are mostly reciprocal, and connectivity between the amygdala and unimodal sensory regions is organized in such a way that efferents arise from the higher order sensory areas, whereas the amygdala targets the primary or secondary sensory areas (Amaral et al. 1992; Turner et al. 1980). Visual input arises specifically from areas of the ventral visual stream, and gustatory and somatosensory information reaches the amygdala through a relay in the insula (Aggleton 1993; Mesulam and Mufson 1985).

The orbitofrontal cortex is involved in the integration of value-related olfactory and gustatory information with viscerosensory information (processed in the anterior insula), and in the transfer of this information to the pACC (Rolls 2019). The lateral orbitofrontal cortex showed a stronger functional connectivity with the gyral components of pACC area p24 (i.e., areas p24a and p24b), whereas medial orbitofrontal areas are more tightly associated with p24c (i.e., the sulcal component of area p24) and with p32 (Palomero-Gallagher et al. 2019). The orbitofrontal cortex, together with subgenual cingulate area 25 (a key node of the cortical autonomic network; Gianaros et al. 2005; Kimmerly et al. 2005; Wong et al. 2007), also modulates autonomic and visceral functions in response to the valence of the stimulus, and does so via connections with the anterior insula, periaqueductal gray and hypothalamus (Critchley and Harrison 2013; Öngür and Price 2000; Palomero-Gallagher et al. 2015; Rempel-Clower and Barbas 1998).

The insula plays a major role in functional integration, and is thought to constitute a correlate of consciousness (Craig 2009). It is a structurally and functionally segregated brain region involved in olfactory, gustatory, sensorimotor and cognitive processes, including emotion processing (Kurth et al. 2010; Mesulam and Mufson 1985). Interestingly, a meta-analysis of functional imaging studies revealed an overlap of activations related to the olfacto-gustatory, emotional and cognitive domains in the anterior-dorsal insula, which thus constitutes a key region in the human brain for the integration of olfaction, emotion and memory (Kurth et al. 2010). Furthermore, activation levels in the anterior insular cortex serve as correlates of the intensity of the experienced emotion, regardless of its valence (Zhu et al. 2019).

The amygdala targets the subgenual and pregenual parts of the anterior cingulate cortex (sACC and pACC, respectively) via the uncinate fasciculus, and cingulate regions are interconnected via the cingulate bundle (Dejerine 1895). The two ACC regions and the anterior midcingulate cortex (aMCC) monitor sensory stimuli, whereby ACC areas monitor emotional stimuli with respect to their pleasantness or unpleasantness (Oane et al. 2020; Palomero-Gallagher et al. 2015, 2019; Vogt and Miller 1983), and areas of the aMCC region play a crucial role in both the perception and anticipation of pain (Porro and Lui 2009; Vogt et al. 1996; Vogt and Sikes 2009b).

It is widely accepted that areas of the sACC subserve the processing of negatively valenced stimuli (Etkin et al. 2011; George et al. 1995; Karama et al. 2011; Liotti et al. 2000; Mechias et al. 2010; Smith et al. 2011). The processing of sadness and fear activates cytoarchitectonic areas s24 and s32, respectively (Palomero-Gallagher et al. 2015). Interestingly, pACC area p32 is associated with the domains of anxiety and fear, though these activations were elicited by tasks requiring the induction of emotions and theory of mind processes, and not by the experience of the emotion itself (Palomero-Gallagher et al. 2015). This association of area p32 with the subject’s ability to experience empathy highlights the unique position of the cingulate cortex as a link between the emotional and memory domains, thus enabling cognitive influences on emotion (Palomero-Gallagher et al. 2015; Stevens et al. 2011). Notably, although some studies studying the neural substrate for the subjective feeling of happiness reported activations within ACC (e.g., Habel et al. 2005; Phillips et al. 1998), no meta-analytic approaches have been able to identify a significant association between the pACC (or any of its areas) and the processing of positively valenced emotions (Kirby and Robinson 2017; Palomero-Gallagher et al. 2015; Phan et al. 2002; Torta and Cauda 2011; Vytal and Hamann 2010).

pACC receives gustatory and viscerosensory input from the orbitofrontal cortex, and is able to integrate visceral sensations via its reciprocal connections with the insula (Qadir et al. 2018; Taylor et al. 2009), and a recent cytoarchitectonically informed meta-analysis found the gyral components of pACC area p24 to be significantly associated with the behavioral domains of gustation and interoception (Palomero-Gallagher et al. 2019). These areas also co-activate with areas of the affective network (George et al. 1995; Lévesque et al. 2003), highlighting the importance of reward value in the generation of emotions (Glascher et al. 2012; Grabenhorst and Rolls 2011). Area p32 of pACC, and also area s24 of sACC, are involved in estimating the emotional valence of faces via visual input arising from areas of the ventral stream (Palomero-Gallagher et al. 2015, 2019). The pACC is also involved in conflict monitoring, and the sulcal component of area p24 is associated with action inhibition, and co-activates with components of the salience network (Palomero-Gallagher et al. 2019). Thus, the pACC integrates information from the dorsolateral prefrontal cortex concerning the selection and maintenance of options to current or proposed behaviors to provide the motivation to carry out selected behavior (Holroyd and Yeung 2012). Furthermore, it was shown that face-evoked responses in the anterior insula and anterior cingulate cortex contain information which is shaped by social interaction, and it was hypothesized that this provides a substrate of how social inclusion shapes future behavior and interaction, while the recognition of individual faces is supported by the visual cortex (Eger et al. 2013).

As part of both the amygdala- and the hippocampus-centered network, the ACC region is also able, either via its direct reciprocal connections with the rostral hippocampus, or in a relay through the thalamus, to modulate the consolidation and retrieval of memory (Aggleton 2012; Navawongse and Eichenbaum 2013; Xu and Sudhof 2013). Given that memories of emotionally valenced stimuli are easier to recollect than those of neutral ones, the ACC is thought to facilitate retrieval of related and competing memories by creating contextual representations of these experiences during the consolidation phase (Bian et al. 2019).

The aMCC receives input from ACC regions and also via the medial pain system and is thus in an ideal position to modulate avoidance behavior in response to noxius stimuli (Vogt 2005), whereby activations were found to be proportional to the degree of pain experienced (Derbyshire et al. 1998; Vogt et al. 1996). The aMCC is also activated during the processing of negatively valenced stimuli, and involved in the expression of fear responses (Pereira et al. 2010; Vogt et al. 2003). The MCC region projects to the supplementary areas, and the sulcal component of aMCC also contains a cingulate motor area (Morecraft and Tanji 2009; Vogt and Sikes 2009b), which projects directly to the facial motor nucleus and to portions of the spinal cord that control finger and hand movements. Thus, a brain network subserving emotion is able to directly generate and modulate facial, limb, or vocal reactions in response to a perceived stimulus. Furthermore, aMCC is thought to coordinate skeletomotor reflex responses in fear avoidance strategies (Vogt et al. 2003).

The ventral striatopallidum encompasses the ventral portions of the caudate nucleus, putamen and globus pallidus, as well as the nucleus accumbens and the olfactory tubercle (Mesulam 2000). It receives direct input from the amygdala, but is also connected with the orbitofrontal cortex and the ACC, and is a central component of the reward circuit, and in the generation of emotional motor activity (Nieuwenhuys et al. 2008).

### The hippocampus-centered network

The hippocampus-centered network mediates the integration of information processed by multiple large-scale brain networks involved in the different memory types to incorporate cognition into emotion processing. It includes the hippocampal complex, entorhinal and retrosplenial cortex (RSC), areas of the anterior (discussed above) and posterior cingulate cortex, as well as the thalamus (Mesulam 2000).

The hippocampal formation is a key structure in the consolidation and retrieval of declarative, spatial and emotional memory (Bird and Burgess 2008; Fanselow and Dong 2010; Strange et al. 2014), and the entorhinal cortex represents the nodal point in neocortico-hippocampal circuits (Insausti and Amaral 2008). The hippocampal formation consists of the hippocampus proper, with the Cornu Ammonis regions CA1–CA4 and the fascia dentata, and the subicular complex, with the prosubiculum, subiculum, presubiculum, and parasubiculum (Palomero-Gallagher et al. 2020). The hippocampus is situated at the top of a highly complex interconnected and hierarchically organized network participating in memory functions (for a comprehensive review see Aggleton 2012), and its reciprocal connections with the amygdala are of particular importance for affective and social learning (Insausti and Amaral 2012; Yilmazer-Hanke 2012).

The dorso-ventral axis of the rodent hippocampus, which is homolog to a posterior-to-anterior axis in primates, is structurally and functionally segregated (Fanselow and Dong 2010; Strange et al. 2014). The dorsal hippocampus is more densely connected with the RSC, mammillary bodies, and anterior thalamus, and is mainly involved in cognitive functions such as navigation and exploration (Fanselow and Dong 2010; Jones and Witter 2007; Moser et al. 1993; Risold and Swanson 1997; Strange et al. 2014; Witter 1993). The ventral hippocampus is more strongly connected to the amygdala, nucleus accumbens and hypothalamus, and is involved in motivated behavior and autonomic responses (Canteras and Swanson 1992; Fanselow and Dong 2010; Groenewegen et al. 1987; Henke 1990; Strange et al. 2014; van Groen and Wyss 1990). The primate hippocampus presents a comparable heterogeneity in structural connectivity, with a rostro-caudal decrease in connectivity with the amygdala, nucleus accumbens and prefrontal cortex, and a rostro-caudal increase in connectivity with the posterior cingulate cortex (PCC) and RSC (Aggleton 2012; Friedman et al. 2002; Fudge et al. 2012; Kobayashi and Amaral 2003, 2007).

In humans, the posterior hippocampus is activated during declarative and spatial memory tasks (Greicius et al. 2003; Maguire et al. 1997). Resting state functional connectivity analyses found the posterior hippocampus to be more highly connected to the RSC and lateral parietal cortex, i.e., areas involved in visuospatial cognition, whereas the anterior hippocampus was more strongly connected to the temporal, orbitofrontal and anterior cingulate cortex, i.e., areas associated with motivational behavior (Adnan et al. 2016; Vogel et al. 2020). There is also evidence of anatomical connectivity between the anterior hippocampus and the fusiform gyrus (Duvernoy 2005), a part of the visual system particularly involved in the identification of faces (Kanwisher and Yovel 2006), words (Cohen and Dehaene 2004) and places (Epstein et al. 1999; Epstein 2008), and single neurons in the human hippocampus have not only been found to respond differentially to faces and objects, but also to respond preferentially to specific emotional expressions (Fried et al. 1997). Interestingly, genes expressed in the posterior hippocampus correlate with cortical regions involved in memory processes, whereas gene expression in the anterior hippocampus correlates with regions involved in emotion (Vogel et al. 2020).

The hippocampal complex and entorhinal cortex are interconnected with the RSC and with PCC area 23, though connections are much denser with the former than with the latter region (Kobayashi and Amaral 2003). RSC is also densely interconnected with areas 24 and 23 of the ACC and PCC, respectively (Kobayashi and Amaral 2003, 2007). The RSC is reciprocally connected to dorsolateral prefrontal areas 9 and 46, and thus constitutes a link between the hippocampus and brain regions involved in executive functions (Kobayashi and Amaral 2003, 2007). It receives early visual input from areas v2 and v4 of the ventral stream and is also interconnected with inferior parietal area 7a (Kobayashi and Amaral 2003, 2007), which mediates visuomotor coordination (Rozzi et al. 2008). Reciprocal connections between the anterior thalamic nucleus and both the hippocampus and RSC facilitate the integration of visual and body-based orientation cues (Miller et al. 2014; Shine et al. 2016), the episodic retrieval of familiar places and objects (Sugiura et al. 2005), and provide an anatomical substrate for fear conditioning processes whereby the RSC is critically involved in tasks during which subjects must form appropriate associations among diverse cues and outcomes to perform optimally (Corcoran et al. 2016; Keene and Bucci 2008a, b). Thus, the RSC is in a position to modulate both the storage and retrieval of spatial and contextual information, in particular that related to fear.

The PCC is primarily involved in visuospatial, sensorimotor and long-term memory functions, and in the framework of emotion processing, plays a role in the assessment of the self-relevance of emotional events and stimuli (Vogt and Laureys 2009a). The PCC has reciprocal connections with sACC (Vogt and Pandya 1987), and is also targeted by the hippocampal complex and the RSC (Kobayashi and Amaral 2003, 2007). Furthermore, the PCC receives input from auditory association areas and has extensive connections with the inferior parietal cortex (Vogt and Pandya 1987) through which it receives input from areas belonging to the dorsal visual stream and involved in movement and spatial orientation (Kravitz et al. 2011; Ungerleider and Mishkin 1982). The convergence of visual and auditory stimuli together with information coded for valence in ACC enable the self-referential processing of stimuli and experiences.

## Lateralization of emotion processing

As with language functions, our first inkling of a possible lateralization in the processing of emotions came from clinical observations of patients with left-brain lesions, since despite severe speech impairment, they retained traces of emotional language (Hughlings-Jackson 1878). Two main models of lateralization have been proposed, based on empirical support from studies in both patients and healthy subjects (Gainotti 2019a): the right-hemispheric dominance hypothesis and the valence lateralization hypothesis.

### Right-hemispheric dominance hypothesis

The right-hemispheric dominance hypothesis proposes that the right half of the brain is dominant for the processing of all emotions, independent of their valence or of the emotional feeling being processed (Borod et al. 1998).

Patients with lesions in the right temporo-parietal region (i.e., Wernicke’s region for language comprehension; Wernicke 1874) perform worse in tasks involving comprehension of the emotion expressed by affective speech than do patients with comparable left-hemispheric lesions (Heilman et al. 1975; Tucker et al. 1977). Right-hemispheric lesions affecting the fusiform face area (Kanwisher and Yovel 2006), impair the patient’s ability to recognize the nature of the emotion conveyed by images of emotional faces (Adolphs et al. 1996). More widespread lesions in the right ventrolateral visual cortex also result in the inability to identify the valence or category of emotions depicted in in images of scenes (DeKosky et al. 1980). Unilateral focal excision of the right parieto-occipital cortex (Kolb and Taylor 1981), or electrical stimulation of right temporal visual-related cortex (Fried et al. 1982) also result in an impaired processing of facial expressions. Patients with right-hemispheric lesions are also unable to identify the valence or category of emotions depicted in in images of scenes (DeKosky et al. 1980). Interestingly, pictures of angry, happy or fearful faces, but not of neutral faces, elicited a right-lateralized activation of the amygdala when presented to a patient with bilateral damage to the primary visual cortex (Pegna et al. 2005). A recent meta-analysis revealed a significant correlation between the degree of emotional impairment in patients suffering from frontotemporal lobar degeneration and the degree of atrophy or hypometabolism of frontotemporal structures in the right hemisphere (Gainotti 2019b). A right-hemispheric dominance has been demonstrated for both the generation and the perception of emotional displays, since facial expressions on the left side of the face are more emotionally intense than those on the right side, and participants perceive emotional expressions to be more emotional when presented in the left than in the right visual field (Blom et al. 2020; Burt and Hausmann 2019; Lindell 2018; Prete et al. 2015; Sackeim and Gur 1978; Sackeim et al. 1978; Wyczesany et al. 2018).

Processing of emotion expression has also been found to be associated with lateralization of white matter pathways. The volumetric asymmetry of the uncinate fascicle, which connects components of the temporo-amygdala-orbitofrontal network and is larger in the right than in the left hemisphere, is positively correlated with lateralization of emotional expressivity of sad faces (Ioannucci et al. 2020). Furthermore, the rightward lateralization of the dorsal component of the superior longitudinal fascicle (i.e., SLF I) is negatively correlated with lateralization of emotional expressivity of happy faces (Ioannucci et al. 2020), and disruptions in the SLF are the most common white matter alteration in patients suffering from psychiatric emotional conditions (Jenkins et al. 2016).

### Valence lateralization hypothesis

According to the valence lateralization hypothesis, both hemispheres are involved in the processing of emotion and emotional feelings, but in a manner dependent on the emotional valence of the information being processed, with a preference of the left hemisphere for positively valenced emotions and of the right one for negatively valenced ones (Davidson 1983). In a variant of this hypothesis, lateralization would be driven by motivational valence, with the left hemisphere being dominant for approach motivational tendencies and the right one for withdrawal ones (Demaree et al. 2005).

This hypothesis was formulated to explain the fact that pathological laughing conditions or indifference to one’s own illness were frequently associated with damage to the right hemisphere, whereas pathological crying or the onset of depressive symptoms occurred mostly in patients with lesions to the left hemisphere (Bear 1983; Sackeim et al. 1982). Further support is provided by the observation that speech with a positively valenced emotional content resulted in an activation of the left amygdala of a long-term unresponsive comatose patient (Eickhoff et al. 2008), and positive visual stimuli elicit a left amygdalar activation in healthy subjects (Canli et al. 1998; Hamann et al. 2002; Lee et al. 2004). Interestingly, this normal left-lateralized amygdalar activity is often disturbed in patients with mental disorders (Allen et al. 2021; Baas et al. 2004). Divided visual field studies revealed that identification of positively valenced facial expressions or emotional words is faster and more accurate when these are presented in the right than in the left hemifield (i.e., when the stimulus is processed by the left vs. right hemisphere), and the opposite holds true for negatively valenced facial expressions or emotional words (Holtgraves and Felton 2011; Jonczyk 2015; Martin and Altarriba 2017; Reuter-Lorenz and Davidson 1981; Wyczesany et al. 2018). Analysis of alpha-band electroencephalographic activity in the frontal lobe revealed that stimuli designed to induce happiness elicit a greater cortical activity the left hemisphere, whereas stimuli designed to evoke negative emotions resulted in a greater cortical activity in the right hemisphere (Jones and Fox 1992; Zhao et al. 2018). A recent study addressing the ecological validity of the valence lateralization hypothesis by means of a mobile EEG recording system to monitor brain activity of romantic partners in their everyday environment also found emotional kisses to be associated with an increased asymmetry index in alpha-band activity of the frontal lobe (Packheiser et al. 2021).

#### Toward a more differentiated picture

Lateralization associated with the processing of anger is best explained by the motivational variant of the valence hypothesis, since behaviourally this negatively valenced emotion is associated with the same kind of response as is happiness, i.e., with a drive toward the stimulus (Carver and Harmon-Jones 2009; Demaree et al. 2005). In line with the expected approach/withdrawal dominance, dichotic listening studies on the perception of sadness or anger through affective prosody revealed a greater involvement of the right hemisphere in the processing of sadness and of the left hemisphere in that of anger (Gadea et al. 2011). Likewise, viewing of angry faces resulted in a higher left prefrontal activity than that when neutral faces were presented (Schutter and Harmon-Jones 2013), as did anger induced experimentally by manipulated insult (Harmon-Jones and Sigelman 2001).

### Looking into the future: the need for hemispheric functional-equivalence hypotheses

Function-location meta-analyses have been applied in an attempt to quantitatively integrate results from multiple studies belonging to a specific cognitive or emotional domain. E.g., in a meta-analysis of over 100 functional magnetic resonance imaging studies addressing the mechanisms underlying processing of emotional faces (Fusar-Poli et al. 2009a, b), the authors first tested regional activation differences for an effect of laterality independently from the valence of stimulus, and found the components of the emotion network to be bilaterally activated, thus providing no support for the right-hemispheric dominance hypothesis. The authors then searched for possible lateralization patterns based on both the motivational and the drive variants of the valence lateralization hypothesis. When testing for the emotional valence of the stimulus, a laterality was only to be induced by the processing of faces expressing negative emotions. However, contrary to what is predicted by the model, the activation was localized in the left hemisphere. Finally, when grouping stimuli according to their corresponding approach/withdrawal category, a left-lateralized activation was found in the inferior frontal gyrus during the processing of faces encoding approach emotions, and right-lateralized activations occurred in the medial frontal and middle frontal gyri during the processing of faces encoding withdrawal emotions. A meta-analysis addressing the neuroanatomical structures underpinning emotional experiences demonstrated that the basic emotions happiness, sadness, fear, anger and disgust are associated with distinct regional brain activation patterns (Vytal and Hamann 2010). A lateralization could only be associated with the processing of fear, since most prominent clusters are located in the right cerebellum and insula, as well as bilaterally in the amygdala. For each of the remaining basic emotions, largest activation clusters were found in both the left and right hemisphere (Vytal and Hamann 2010). Specifically, happiness is associated with activations in the right superior temporal gyrus and the left anterior cingulate cortex, sadness with clusters in the left caudate nucleus and medial frontal gyrus, as well as in the right inferior frontal gyrus. Anger is associated with activations of the left inferior frontal gyrus and right parahippocampal gyrus, and disgust with bilateral insular activations (Vytal and Hamann 2010). Finally, results of a multi-center study evaluating functional connectivity in resting state functional magnetic resonance imaging scans from over a thousand subjects also highlight the existence of both left- and right-dominant intrinsic connectivity hubs rather than that of a global hemispheric lateralization in the human brain (Nielsen et al. 2013). In this context, it has been postulated, that the right-hemispheric dominance and the valence lateralization models may reflect different aspects of emotion processing, thus highlighting the need to move away from the concept of an *overall hemispheric* specialization and to elaborate on the hypothesis that emotions are the result of activations in networks which are interrelated, but may have differential lateralization patterns (Fusar-Poli et al. 2009a; Killgore and Yurgelun-Todd 2007; Neumann et al. 2008).

Along such lines of argument, a hemispheric functional-equivalence hypothesis has recently been formulated to explain lateralization associated with the perception of emotional and neutral faces (Stankovic 2021). It is a dynamic model proposing the existence of an initial default setting in which the brain would be right-biased in emotional and neutral face perception, and this lateralization pattern would be maintained as long as environmental task demands remain low. However, since emotion perception should be viewed as a multi-layered phenomenon, increasing task demands would result in a redistribution of activity among the hemispheres as an adaptive mechanism to ensure continued accurate and prompt responses (Stankovic 2021). Since environmental requirements are known to modulate psychological modulators, this hypothesis would also explain how altered conditions such as acute stress could even result in a reversed lateralization. By proposing the functional-equivalence of both hemispheres, the model also accounts for intersubject variability in lateralization patterns, as it has been demonstrated that not all individuals display the asymmetry predispositions identified at the population level (Frasnelli and Vallortigara 2018).

Finally, a recent data-driven meta-analysis revealed that the perception, experience and expression of emotion are each subserved by a distinct large-scale network (Morawetz et al. 2020). Furthermore, three of these networks are composed of left-lateralized of bilaterally activated areas, whereas the fourth one contains left-lateralized, right-lateralized and bilateral activations. This is particularly interesting, given that the hemispheric functional-equivalence hypothesis of emotional face perception assumes an initial right-biased lateralization (Stankovic 2021), whereas the network that Morawetz et al. (2020) found to be associated with the perception of emotion (albeit not specifically in facial expressions) exhibits left-lateralized or bilateral activations. It thus appears necessary to not only abandon hypotheses supporting the concept of an *overall hemispheric* specialization, but to also move away from a *global model* of lateralization in emotion processing.
